# Importance of the 2,6-Difluorobenzamide Motif for FtsZ Allosteric Inhibition: Insights from Conformational Analysis, Molecular Docking and Structural Modifications

**DOI:** 10.3390/molecules28052055

**Published:** 2023-02-22

**Authors:** Thibaut Barbier, Oana Dumitrescu, Gérard Lina, Yves Queneau, Laurent Soulère

**Affiliations:** 1 Univ Lyon, INSA Lyon, Université Claude Bernard Lyon 1, CNRS, CPE-Lyon, ICBMS, UMR 5246, Institut de Chimie et de Biochimie Moléculaires et Supramoléculaires, Bâtiment Lederer, 1 Rue Victor Grignard, 69622 Villeurbanne, France; 2 Hospices Civils de Lyon, Hôpital de la Croix Rousse-Centre de Biologie Nord, Laboratoire de Bactériologie, Institut des Agents Infectieux, Grande Rue de la Croix Rousse, 69004 Lyon, France; 3 Team StaPath, CIRI, Centre International de Recherche en Infectiologie, Inserm, U1111, Université Claude Bernard Lyon 1, CNRS, UMR 5308, ENS de Lyon, 69007 Lyon, France

**Keywords:** conformational analysis, 2,6-difluorobenzamide, FtsZ, *S. aureus*

## Abstract

A conformational analysis and molecular docking study comparing 2,6-difluoro-3-methoxybenzamide (DFMBA) with 3-methoxybenzamide (3-MBA) has been undertaken for investigating the known increase of FtsZ inhibition related anti *S. aureus* activity due to fluorination. For the isolated molecules, the calculations reveal that the presence of the fluorine atoms in DFMBA is responsible for its non-planarity, with a dihedral angle of -27° between the carboxamide and the aromatic ring. When interacting with the protein, the fluorinated ligand can thus more easily adopt the non-planar conformation found in reported co-crystallized complexes with FtsZ, than the non-fluorinated one. Molecular docking studies of the favored non-planar conformation of 2,6-difluoro-3-methoxybenzamide highlights the strong hydrophobic interactions between the difluoroaromatic ring and several key residues of the allosteric pocket, precisely between the 2-fluoro substituent and residues Val203 and Val297 and between the 6-fluoro group and the residues Asn263. The docking simulation in the allosteric binding site also confirms the critical importance of the hydrogen bonds between the carboxamide group with the residues Val207, Leu209 and Asn263. Changing the carboxamide functional group of 3-alkyloxybenzamide and 3-alkyloxy-2,6-difluorobenzamide to a benzohydroxamic acid or benzohydrazide led to inactive compounds, confirming the importance of the carboxamide group.

## 1. Introduction

**Figure 1:**
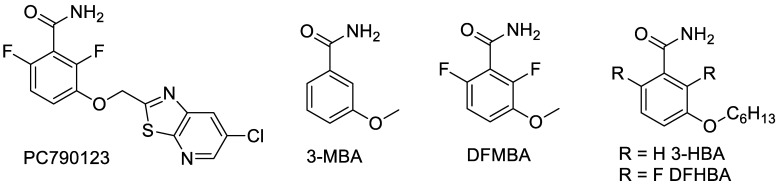
Structure of benzamide FtsZ inhibitors: PC190723, 3-MBA, DFMBA, 3-HBA and DFHBA.

Filamentous temperature-sensitive protein Z (FtsZ) plays a major role in bacterial division like tubulin in eukaryotic cells [1]. The Z ring is required for this process and is formed by the FtsZ recruitment and polymerization [2,3]. Subsequently the contraction of the Z ring is leading to the separation of two cells. As a key protein of the bacterial divisome, the FtsZ protein is extensively studied either for the biological understanding of the cell division process in different species but also to interfere with bacterial growth to develop new antibiotics [4,5]. For this purpose, different strategies are commonly investigated such as the design of FtsZ-ZipA interactions inhibitors, or FtsZ inhibitors targeting the GTP binding site or other allosteric pockets [6,7,8,9]. The latter approach is probably the most studied and the discovery of the benzamide scaffold or more precisely the 2,6-difluorobenzamide nucleus has offered new opportunities in the development of FtsZ allosteric inhibitors [10]. The compounds 2,6-difluoro-3-methoxybenzamide (DFMBA), for which full crystallographic data of its complex with FTSZ are available [11], has been indeed found to be more active as antibacterial agent against *S. aureus* than the 3-methoxybenzamide (3-MBA) and they have been both shown to target FtsZ [12,13]. Consistent effects of fluorination are reported also for the even more active hexyloxy compounds 3-HBA and DFHBA [14,15,16]. The 2,6-difluorobenzamide PC790123, which was studied through Molecular Dynamics in complex with the FtsZ protein [17] is another example of such inhibitor, usually employed as a reference compound in FtsZ inhibition studies (Figure 1) [17,18]. Using benzamide fluorescent probes, the molecular mechanism of the inhibition was suggested through the binding of benzamide inhibitors into the open clefts in cellular FtsZ polymers preferably to free cytosolic FtsZ [19].

Importance of fluorination in medicinal chemistry is now well recognized for improving metabolic stability and also to induce several possible effects, either electronic, steric or polar vs non-polar interactions [20,21,22].

After having focused on the azole moiety of tripartite benzamide inhibitors of FtsZ [23], we now report our studies focusing on the fluorobenzamide moiety. The work reported herein aimed at understanding why this difluorobenzamide motif is so important, through conformational and molecular docking studies investigating 3-MBA and DFMBA for which co-crystallographic with FtsZ data are known. In addition, slight structural variations of the carboxamide functional group, such as benzohydroxamic acid or benzohydrazide were investigated.

## 2. Results and Discussion

**Figure 2:**
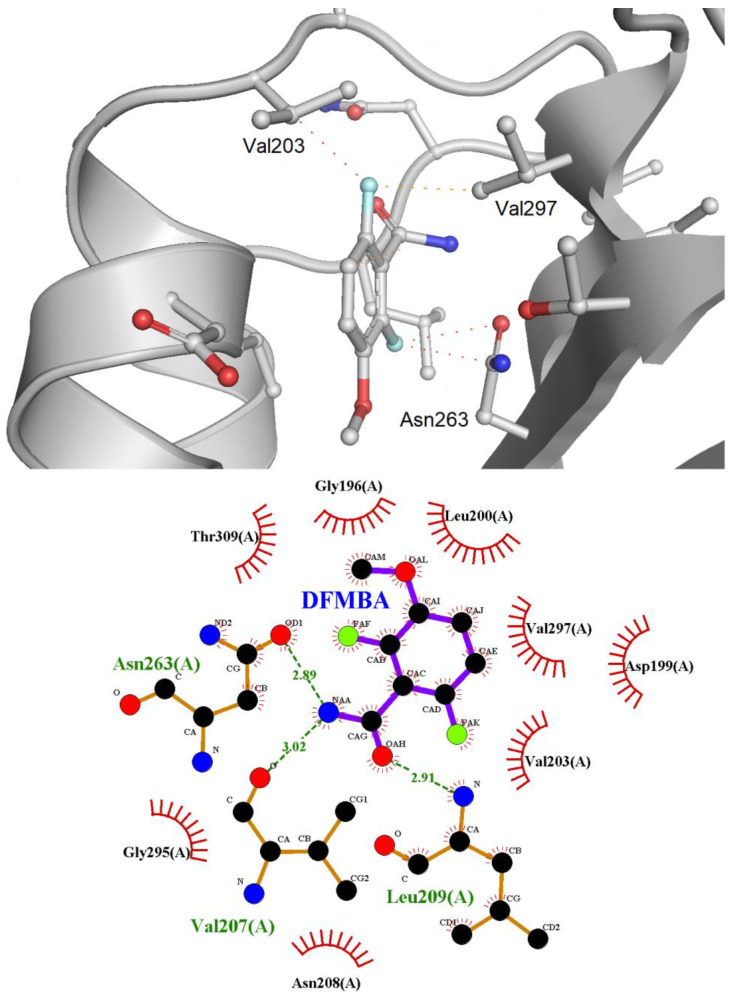
Visualization of the interactions revealed by the Arpeggio server [24] indicated in red dashes of the fluorine atoms of the benzamide nucleus with residues of the FtsZ protein and 2D representation of DFMBA in the allosteric pocket (PDB: 6YD1 [11]).

The benzamide scaffold is extensively used to develop antibacterial agents in relation with the ability to inhibit FtsZ, with significant increased activity when fluorinated. This difference of can be explained by hydrophobic interactions of the fluorine atoms of the benzamide nucleus with the allosteric site of the FtsZ proteins. In order to visualize these interactions, the complex between DFMBA and the staphylococcal FtsZ [11] was submitted to the Arpeggio webserver [24] which monitors all interactions between a ligand and a protein (Figure 2). In addition to three hydrogen bonds between the carboxamide functional group and the residues Val207, Leu209 and Asn263, the difluorobenzamide motif develops key interactions via hydrophobic interactions [25] between the 6-fluoro substituent and the central CH of the isopropyl group of the residue Val203 with (distance F→C of 3.3 Å) and also with the terminal methyl group of the residue Val297 (distance F→C of 2.9 Å). The 2-fluoro substituent interacts with the residues Asn263 via C-F/C=ONH_2_ interactions [20] with a F→C=O distance of 3.2 Å and a F→O=C distance of 3.0 Å.

**Figure 3:**
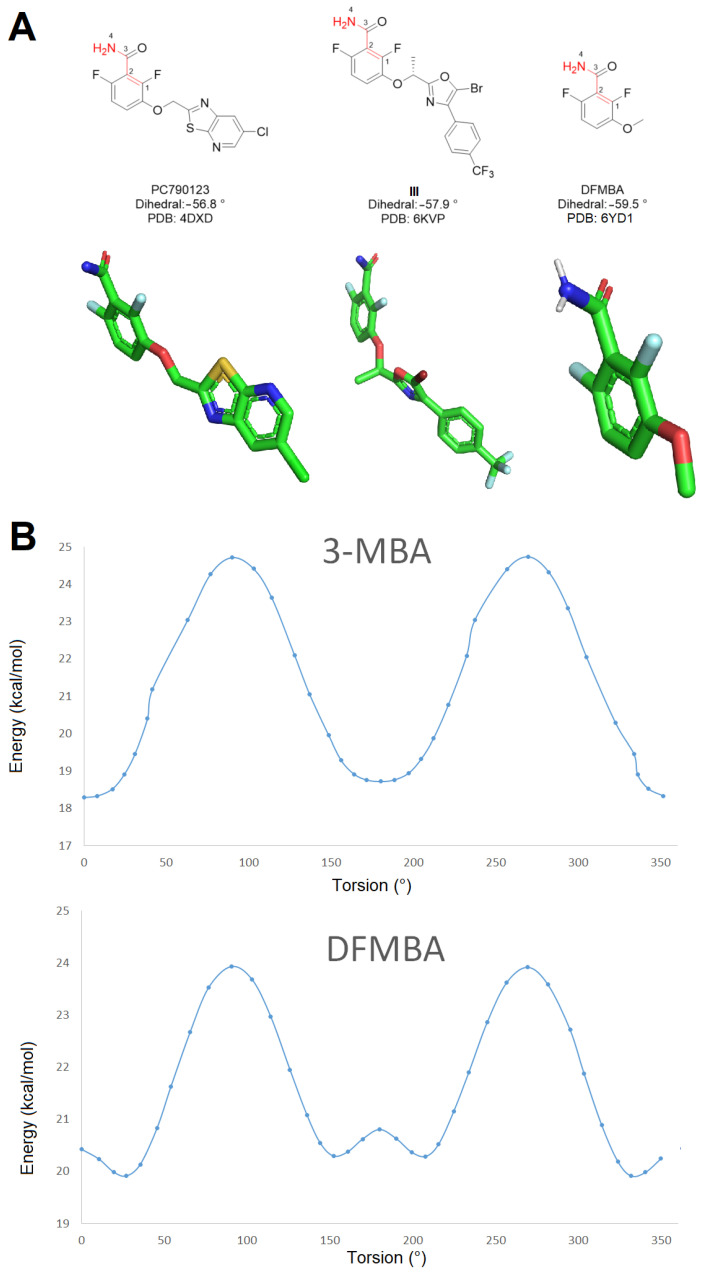
(**A**) Conformations of PC190723 [26] and other 2,6-difluorobenzamide derivatives (DFMBA and compound **III**) [11,28] co-crystallized with FtsZ. (**B**) Conformational analysis of the 3-MBA and DFMBA resulting from a systematic conformational search by varying the torsion defined by the rotation around the bond between the amide functional group and the aromatic ring (increment of 10° and subsequent minimization).

To further investigate the difluorobenzamide nucleus, conformational analysis of the 3-methoxybenzamide 3-MBA and the 2,6-difluoro-3-methoxybenzamide DFMBA was then performed to determine their preferential conformations. A first important observation is that the co-crystallized structure of PC190723 in FtsZ, [26,27] like other difluorobenzamide derivatives [11,28] within the allosteric pocket of the protein shows that the difluorobenzamide nucleus is not planar, though conjugated (Figure 3A). This is also the case for other 2,6-difluorobenzamide-based ligands co-crystallized with FtsZ. For this purpose, a systematic conformational search [29] with an increment of 10° was applied using VEGA ZZ [30,31] on the dihedral angle defined by the rotation around the bond between the amide functional group and the aromatic ring. As depicted in Figure 3B and as expected the preferential conformation of the conjugated benzamide of 3-MBA is a planar conformation with angle values of 0° or 180° allowing the conjugation. For the 2,6-difluorobenzamide scaffold of DFMBA, the curve resulting from the conformational analysis reveals that conformations with the lowest energy are not planar, but with a torsion angle value of −27°. 

These results contrast with the conjugated benzamide scaffold, for which the most stable conformations are planar. Therefore the torsion angle value of −27° due to the presence of the fluorine atoms is closer to the value of the same torsion measured in co-crystallized ligand (−58°, Figure 3A). Moreover, the presence of the fluorine atoms, which did not allow conjugation of the π system, reduces the energetic differences of the bound versus unbound ligand. Indeed, the energetic gap to reach a dihedral angle of −58° from the most stable conformation is 3.71 kcal/mol for benzamide (360° → 302°) while only 1.98 kcal/mol for 2,6-difluorobenzamide (333° → 302°). As a consequence, fluorinated benzamide derivatives, which are now the common pattern of FtsZ allosteric inhibitors may also be considered as conformationally restrained inhibitors [32] showing more potency than their non-fluorinated counterparts. Consistent angles were measured for the hexyl derivatives 3-HBA and DFHBA (data shown in Supplementary Materials see Figure S1).

**Figure 4:**
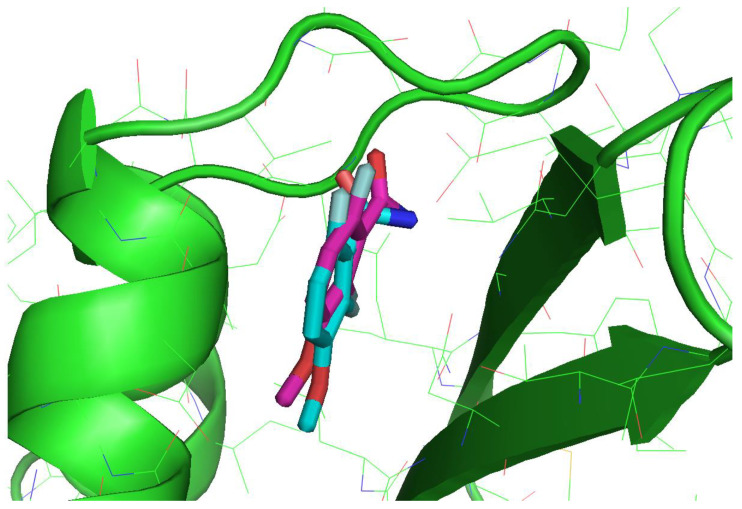
Binding mode of DFMBA obtained as a result of docking simulations (magenta) superimposed with the co-crystallized ligand (cyan).

The preferential conformation of DFMBA with a torsion angle value of −27° was docked as rigid ligand within the allosteric site of *Sa*FtsZ in order to compare the binding mode with the co-crystallized ligand (Figure 4). Both conformations, the one with a torsion angle value of −27° and the co-crystallized ligand (with a torsion angle value of −58°) are close.

**Figure 5:**
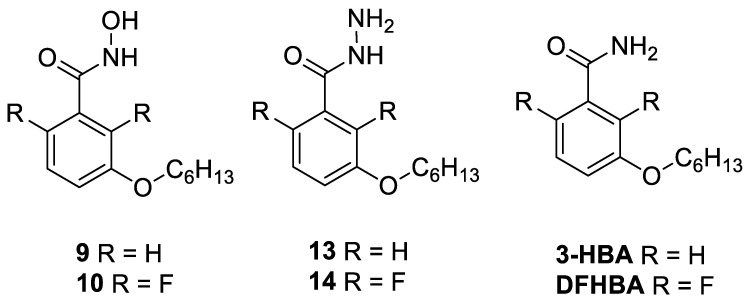
Structure of the known FtsZ inhibitors 3-HBA and DFHBA and of analogues **9**, **10**, **13**, **14**.

A short investigation of structural analogs in which the benzamide group is changed to isosteric groups [33] benzohydroxamic acids and benzohydrazides was then conducted, with either non-fluorinated or fluorinated phenyl groups, and comparison to the know FtsZ inhibitors 3-hexyloxybenzamide (3-HBA) and 3-hexyloxy-2,6-difluorobenzamide (DFHBA) used here as model compounds exerting cell division inhibition of *B. subtilis* [14] with higher activity compared to the methyloxybenzamide derivative (Figure 5) [15]. DFHBA is also know to induce morphological modifications of *B. subtilis* and *S. aureus* bacteria observed by morphometric analysis [14].

**Figure 6:**
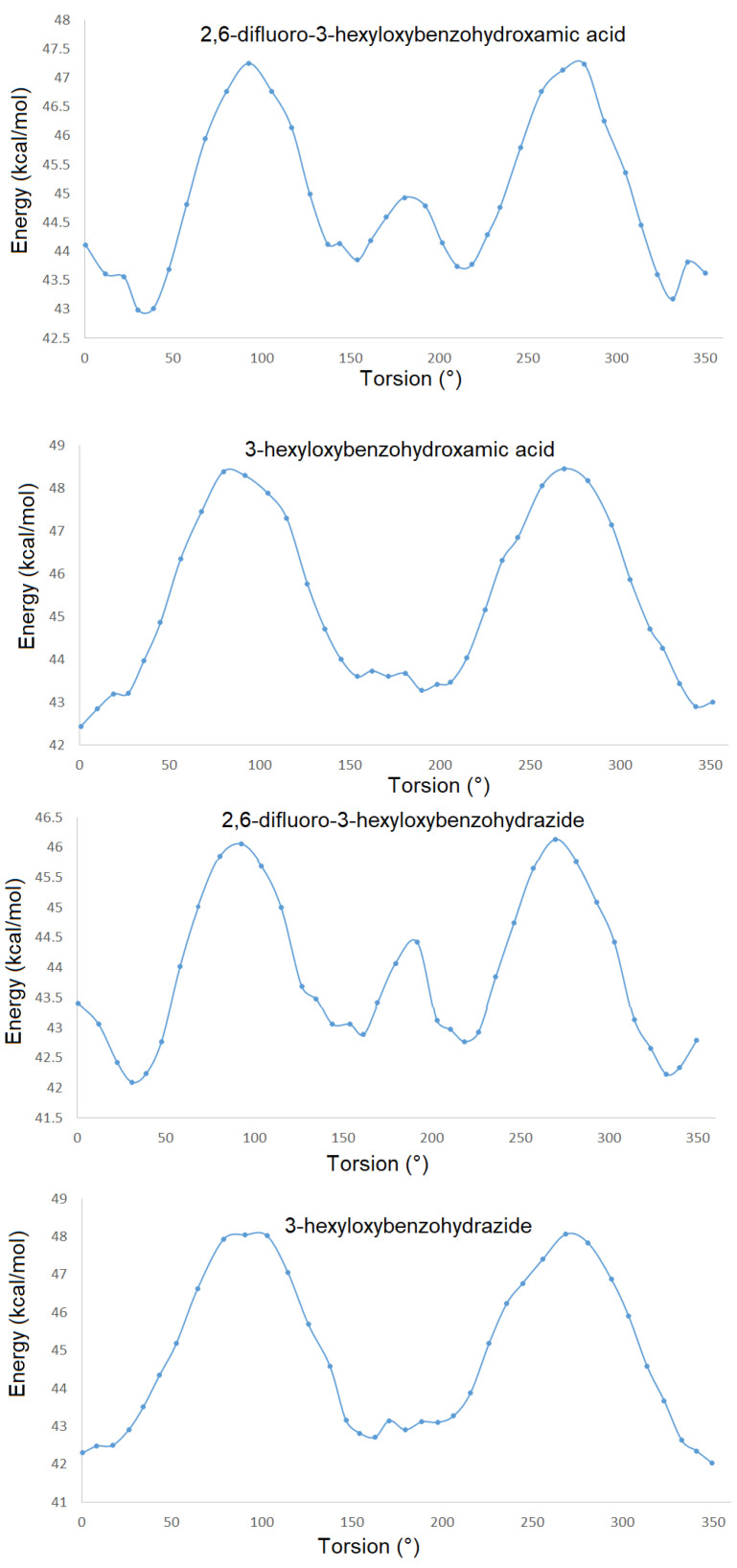
Conformational analysis of fluorinated or non-fluorinated 3-hexyloxy-benzohydroxamic acid and 3-hexyloxy-benzohydrazide.

With respect to their conformation, **9**, **10**, **13**, **14** show comparable behavior to that of 3-HBA and DFHBA, with the fluorine atoms inducing nonplanar stable conformations in contrast to those adopted by non-fluorinated compounds (Figure 6). A torsion angle value of −30° was obtained for the most stable conformations for fluorinated benzohydroxamic acid and benzohydrazides, which is comparable to DFMBA and DFHBA.

**Scheme 1:**
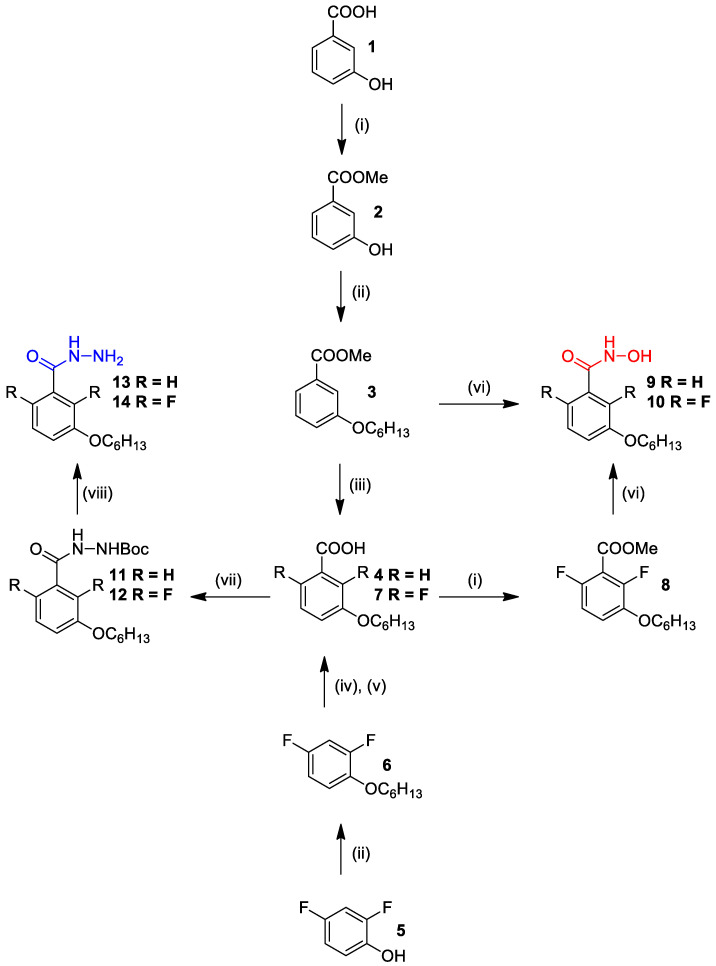
Synthesis of non-fluorinated or fluorinated analogues. Reagents and conditions: (i) SOCl_2_, MeOH, 0 °C then 65 °C, 3 h (**2**: 88%; **8**: 84%); (ii) hexyl bromide, K_2_CO_3_, NaI, ACN, 70 °C, 7 h (**3**: 58%; **6**: 77%); (iii) NaOH, EtOH, r.t., 16 h (**4**: 93%); (iv) n-BuLi, THF, −78 °C, 1 h; (v) dry ice, THF, r.t., 2 h (**7**: 58%); (vi) hydroxylamine hydrochloride, NaOH, H_2_O, dioxane, 0 °C then r.t., 16 h (**9**: 36%; **10**: 47%); (vii) *tert*-butylcarbazate, DCC, DMAP, DCM, 0 °C then r.t., 16 h (**11**: 64%; **12**: 52%); (viii) TFA, DCM, r.t., 24 h (**13**: 41%; **14**: 94%).

With respect to the biological activity, the four analogs **9, 10, 13, 14** were evaluated as antimicrobial agents against three *S. aureus* strains, and compared with compounds 3-HBA and DFHBA used as references. The non-fluorinated and fluorinated 3-hexyloxy-2,6-difluorobenzamide analogues were synthesized as depicted in Scheme 1. 3-HBA and DFHBA were synthesized as previously reported from the compounds 3-hydroxybenzamide and 3-hydroxy-2,6-difluorobenzamide which were alkylated with 1-bromohexane [14,15].

3-(Hexyloxy) benzohydroxamic acids **9** and **10** were prepared from the corresponding methyl esters **3** and **8** and hydroxylamine in basic conditions. The ester **3** was synthesized starting from 3-hydroxybenzoic acid **1** which was converted to its methyl ester counterpart with thionyl chloride in dry methanol followed by the alkylation of the phenol functional group with hexylbromide using potassium carbonate. The ester **8** was obtained starting from the 2,4-difluorophenol which was alkylated with hexylbromide followed by an ortho-lithiation and subsequent carboxylation in the presence of dry ice to give the acid **7**. The latter was converted to the methyl ester with thionyl chloride in dry methanol. The benzohydrazides **13** and **14** were prepared from the non-fluorinated and fluorinated acids **4** and **7** through a standard coupling reaction with *tert*-butyl carbazate and DCC and subsequent deprotection in acidic conditions.

The antimicrobial activity was assessed by determining the corresponding MIC values against three *S. aureus* strains: a reference strain (ATCC29213), a methicillin-resistant strain (SF8300) [34] and a clinically isolated daptomycin-resistant strain (ST20171643) [35] (Table 1). 3-HBA and DFHBA, used as references, exert MIC values of 256 µg/mL and 8 µg/mL consistent with the literature [14,15,16]. For the benzohydroxamic acid or benzohydrazides, either fluorinated of non-fluorinated, the activity is at least decreased to 64 µg/mL.

The modification of the benzamide motif is therefore highly detrimental to the activity, consistently with previous other attempts such as with a substitution to a sulfonamide group [36]. As demonstrated in this study, the 2,6-difluorobenzamide motif could be considered as both, a conformational restrained scaffold [32] with fluorine acting as conformational control element and an optimized structure to develop hydrophobic and C-F/C=O interactions as well as several hydrogen bonds.

## 3. Experimental

### 3.1. Conformational Analysis

The compounds DFMBA, 3-MBA and analogues were drawn using VEGA ZZ [30,31] and were minimized using the SP4 force field with Gasteiger charges. Conformational analyses were carried out using the conformational search module by varying the torsion angle defined by the bond between the benzamide, the hydroxamic acid or the hydrazide and the aromatic ring using a 10° increment and minimization of each generated conformers. Subsequently to the conformational analyses, energy graphs were created to analyze the conformation landscape of the compounds and to find preferential conformations.

### 3.2. Molecular Docking

Subsequently to the conformational analysis, the preferential conformation of the DFMBA with a torsion angle value of −27° was retrieved and a rigid ligand docking was performed on *Staphylococcus aureus Sa*FtsZ (PDB: 6YD1) with Arguslab [37] with a docking box of 15 Å × 15 Å × 15 Å centered on the ligand using the genetic algorithm with default parameters. The docking result was superimposed to the structure of the co-crystallized DFMBA (PDB: 6YD1). 3D-Visualization was performed with PyMOL which was used to create Figure 1 and Figure 4. 2D-representation was created with LigPlot+ [38] for Figure 1.

### 3.3. Chemistry

All commercial materials were used as received without further purification. Flash chromatography was carried out using Macherey–Nagel Kieselgel 60 M silica. Analytical thin-layer chromatography was realized using aluminum-backed plates coated with Macherey–Nagel Kieselgel 60 XtraSIL G/UV254 and were visualized under UV light (at 254 nm or 365 nm) or stained using ninhydrin. Nuclear magnetic resonance (NMR) spectra were recorded on Bruker AV300, AV400 or Bruker AV500 spectrometers, operating at 300 MHz, 400 MHz and 500 MHz, respectively, for the proton (^1^H) NMR and at 75 MHz, 100 MHz and 125 MHz, respectively, for the carbon (^13^C) NMR. Chemical shifts were reported in parts per million (ppm) on a scale relative to residual solvent signals. Multiplicities are abbreviated as: s, singlet; d, doublet; t, triplet; q, quadruplets; dd, doublet of doublets; dt, doublet of triplets; td, triplet of doublets; ddd, doublet of doublet of doublets; m, multiplet. Coupling constants were measured in Hertz (Hz). High-resolution mass spectra (HRMS) and low-resolution mass spectra were obtained by the Centre Commun de Spectrométrie de Masse (CCSM), University of Lyon 1, Lyon, France. 3-HBA and DFHBA were synthesized by alkylation of 3-hydroxybenzamide and 2,6-difluoro3-hydroxybenzamide with 1-bromohexane as previously reported [14,15].

#### 3.3.1. (i) General Procedure for Methyl 3-hydroxybenzoate

To a solution of 3-hydroxybenzoic acid (15.0 mmol, 1.0 eq.) in MeOH (20 mL) at 0 °C was added dropwise thionyl chloride (75.0 mmol, 5.0 eq.). The solution was stirred at 65 °C for 3 h and then cooled to room temperature. The solvent was evaporated and the residue retaken in saturated NaHCO_3_ solution, extracted with EtOAc, dried over Na_2_SO_4_ and concentrated under vacuum to give desired product.

##### Methyl 3-Hydroxybenzoate (2)

White powder (2.014 g, 88% yield). ^1^H NMR (300 MHz, Acetone-*d_6_*) δ 8.67 (s, 1H), 7.52–7.46 (m, 2H), 7.33 (ddd, *J* = 8.1, 7.2, 0.9 Hz, 1H), 7.09 (ddd, *J* = 8.1, 2.4, 1.3 Hz, 1H), 3.86 (s, 3H). (Spectrum in accordance with Gill et al., 2021 [39]).

##### Methyl 2,6-Difluoro-3-(hexyloxy)benzoate (8)

Orange oil (387 mg, 84% yield). ^1^H NMR (300 MHz, Chloroform-*d*) δ 6.99 (td, *J* = 9.1, 5.1 Hz, 1H), 6.82 (td, *J* = 9.1, 2.0 Hz, 1H), 3.97 (t, *J* = 6.5 Hz, 2H), 3.93 (s, 3H), 1.82–1.69 (m, 2H), 1.50–1.36 (m, 2H), 1.36–1.24 (m, 4H), 0.93–0.81 (m, 3H); ^13^C NMR (75 MHz, Chloroform-*d*) δ 162.14, 153.77 (dd, *J* = 249.0, 4.9 Hz), 150.64 (dd, *J* = 257.2, 6.4 Hz), 144.05 (dd, *J* = 10.8, 3.5 Hz), 117.76 (dd, *J* = 9.6, 3.5 Hz), 111.79 (dd, *J* = 19.8, 15.9 Hz), 110.89 (dd, *J* = 22.9, 4.4 Hz), 70.59, 52.85, 31.56, 29.21, 25.59, 22.63, 14.04; MS (ESI) m/z = 273.1 [M+H]^+^, 295.1 [M+Na]^+^.

#### 3.3.2. (ii) General Procedure for Methyl 3-(hexyloxy)benzoate

To a solution of methyl 3-hydroxybenzoate (13.2 mmol, 1.1 eq.) in acetonitrile (15 mL) were added 1-bromohexane (1.6 mmol, 1.0 eq.), K_2_CO_3_ (20.1 mmol, 1.6 eq.) and NaI (1.3 mmol, 0.10 eq.). The solution was stirred at 70 °C for 7 h. After evaporation of the solvent, the residue was diluted with water, extracted with Et_2_O, dried over Na_2_SO_4_ and concentrated under vacuum to give desired product.

##### Methyl 3-(hexyloxy)benzoate (3)

Translucent oil (1.719 g, 58% yield). ^1^H NMR (300 MHz, Acetone-*d*_6_) δ 7.57 (ddd, *J* = 7.6, 1.6, 1.1 Hz, 1H), 7.52–7.50 (m, 1H), 7.40 (ddd, *J* = 8.2, 7.6, 0.5 Hz, 1H), 7.18 (ddd, *J* = 8.2, 2.7, 1.1 Hz, 1H), 4.05 (t, *J* = 6.5 Hz, 2H), 3.87 (s, 3H), 1.86–1.72 (m, 2H), 1.56–1.43 (m, 2H), 1.40–1.32 (m, 4H), 0.95–0.87 (m, 3H). (Spectrum in accordance with Cavedon et al., 2019 [40])

##### 2,4-Difluoro-1-(hexyloxy)benzene (6)

Translucent oil (1.648 g, 77% yield). ^1^H NMR (300 MHz, Acetone-*d*_6_) δ 7.14 (td, *J* = 9.3, 5.4 Hz, 1H), 7.04 (ddd, *J* = 11.5, 8.7, 3.0 Hz, 1H), 6.96–6.86 (m, 1H), 4.05 (t, *J* = 6.5 Hz, 2H), 1.86–1.70 (m, 2H), 1.55–1.42 (m, 2H), 1.39–1.29 (m, 4H), 0.94–0.86 (m, 3H); ^13^C NMR (75 MHz, Acetone-*d*_6_) δ 157.02 (dd, *J* = 239.3, 10.5 Hz), 153.21 (dd, *J* = 247.5, 12.2 Hz), 144.83 (dd, *J* = 10.7, 3.4 Hz), 116.61 (dd, *J* = 9.5, 3.1 Hz), 111.26 (dd, *J* = 22.3, 4.1 Hz), 105.28 (dd, *J* = 27.2, 22.4 Hz), 70.57, 32.28, 29.94, 26.33, 23.27, 14.28; HRMS (EI) m/z: calcd. for C_12_H_16_F_2_O [M]^+.^ 214.1164, found 214.1162.

#### 3.3.3. (iii) Procedure for 3-(hexyloxy)benzoic Acid

To a solution of methyl 3-(hexyloxy)benzoate (6.9 mmol, 1.0 eq.) in EtOH (10 mL) was added a solution of NaOH 2N (13.9 mmol, 2.0 eq.). The solution was stirred at room temperature for 16 h, concentrated under vacuum, diluted with H_2_O, acidified with HCl 1 N, extracted with EtOAc, dried over Na_2_SO_4_ and concentrated under vacuum to give desired product.

##### 3-(Hexyloxy)benzoic Acid (4)

White powder (1.437 g, 93% yield). ^1^H NMR (300 MHz, Acetone-*d*_6_) δ 11.22 (s, 1H), 7.61 (ddd, *J* = 7.6, 1.5, 1.0 Hz, 1H), 7.55 (dd, *J* = 2.7, 1.5 Hz, 1H), 7.41 (ddd, *J* = 8.2, 7.6, 0.4 Hz, 1H), 7.18 (ddd, *J* = 8.2, 2.7, 1.0 Hz, 1H), 4.06 (t, *J* = 6.5 Hz, 2H), 1.86–1.74 (m, 2H), 1.56–1.43 (m, 2H), 1.40–1.29 (m, 4H), 0.95–0.85 (m, 3H); ^13^C NMR (75 MHz, Acetone-*d*_6_) δ 167.57, 160.19, 132.76, 130.39, 122.57, 120.26, 115.81, 68.79, 32.31, 29.90, 26.41, 23.27, 14.30; MS (ESI) m/z = 223.3 [M+H]^+^, 245.2 [M+Na]^+^.

#### 3.3.4. (iv), (v) Procedure for 2,6-difluoro-3-(hexyloxy)benzoic Acid

To a solution of 2,4-difluoro-1-(hexyloxy)benzene (7.4 mmol, 1.0 eq.) in THF (15 mL) under a dried and inert atmosphere (N_2_) at −78 °C was added dropwise *n*-BuLi (1.6 M in THF, 8.1 mmol, 1.1 eq.). The solution was stirred at −78 °C for 1 h. The mixture was then added dropwise on crushed dry ice, and the solution was stirred at room temperature for 2 h. To the mixture was then added NaOH solution (2 M, 11 mL) and was extracted with Et_2_O to remove unreacted reagent. The solution was then acidified with an HCl 2 M solution, and the product was extracted with EtOAc, dried over Na_2_SO_4_ and concentrated under vacuum to give the desired product.

##### 2,6-Difluoro-3-(Hexyloxy)Benzoic Acid (7)

Orange oil (1.096 g, 58% yield). ^1^H NMR (300 MHz, Acetone-*d*_6_) δ 10.34 (s, 1H), 7.24 (td, *J* = 9.2, 5.2 Hz, 1H), 6.99 (td, *J* = 9.2, 2.0 Hz, 1H), 4.06 (t, *J* = 6.5 Hz, 2H), 1.77 (dq, *J* = 8.2, 6.5 Hz, 2H), 1.52–1.40 (m, 2H), 1.36–1.27 (m, 4H), 0.92–0.82 (m, 3H); ^13^C NMR (75 MHz, Acetone-*d*_6_) δ 162.53, 153.59 (dd, *J* = 245.9, 5.5 Hz), 149.99 (dd, *J* = 254.1, 6.9 Hz), 144.92 (dd, *J* = 10.7, 3.3 Hz), 117.93 (dd, *J* = 9.6, 3.3 Hz), 113.39 (dd, *J* = 21.3, 16.9 Hz), 111.79 (dd, *J* = 22.8, 4.3 Hz), 70.73, 32.24, 29.87, 26.27, 23.25, 14.29; MS (ESI) m/z = 259.1 [M+H]^+^, 281.1 [M+Na]^+^.

#### 3.3.5. (vi) General Procedure for 3-(hexyloxy)-*N*-hydroxybenzamide

To a solution of hydroxylamine hydrochloride (1.7 mmol, 2.0 eq.) in water (3 mL) at 0 °C was added NaOH (4.25 mmol, 5.0 eq.) and dropwise methyl 3-(hexyloxy)benzoate (0.85 mmol, 1.0 eq.) dissolved in 1,4-dioxane (2 mL). The solution was stirred at room temperature for 16 h. 1,4-dioxane is then removed under vacuum and the mixture is diluted with water, acidified to pH 6 with HCl 2 M and filtered. The obtained solid is then purified by chromatography (eluent: EtOAc/pentane 3:1).

##### 3-(Hexyloxy)-*N*-hydroxybenzamide (9)

Orange powder (72 mg, 36% yield). ^1^H NMR (500 MHz, Methanol-*d*_4_) δ 7.38–7.25 (m, 3H), 7.08–7.02 (m, 1H), 3.99 (t, *J* = 6.4 Hz, 2H), 1.81–1.71 (m, 2H), 1.52–1.42 (m, 2H), 1.42–1.30 (m, 4H), 0.95–0.88 (m, 3H); ^13^C NMR (126 MHz, Methanol-*d*_4_) δ 167.99, 160.72, 134.72, 130.72, 120.01, 119.16, 113.88, 69.18, 32.72, 30.27, 26.81, 23.65, 14.37; HRMS (ESI) m/z: calcd. for C_13_H_20_NO_3_ [M+H]^+^ 238.1438, found 238.1438.

##### 2,6-Difluoro-3-(hexyloxy)-*N*-hydroxybenzamide (10)

Orange powder (77 mg, 47% yield). ^1^H NMR (300 MHz, Methanol-*d*_4_) δ 7.18 (td, *J* = 9.3, 5.2 Hz, 1H), 6.95 (td, *J* = 8.9, 2.0 Hz, 1H), 4.03 (t, *J* = 6.4 Hz, 2H), 1.86–1.70 (m, 2H), 1.54–1.43 (m, 2H), 1.43–1.26 (m, 4H), 0.96–0.88 (m, 3H); ^13^C NMR (126 MHz, Methanol-*d*_4_) δ 160.00, 154.22 (dd, *J* = 243.3, 5.7 Hz), 150.76 (dd, *J* = 251.5, 7.3 Hz), 145.21 (dd, *J* = 10.8, 3.3 Hz), 118.00 (dd, *J* = 9.2, 3.0 Hz), 114.04 (dd, *J* = 23.9, 19.5 Hz), 111.84 (dd, *J* = 22.8, 4.1 Hz), 71.24, 32.66, 30.23, 26.67, 23.63, 14.35; HRMS (ESI) m/z: calcd. for C_13_H_18_F_2_NO_3_ [M+H]^+^ 274.1249, found 274.1253.

#### 3.3.6. (vii) General Procedure for *Tert*-Butyl 2-(3-(hexyloxy)benzoyl)hydrazine-1-Carboxylate

To a solution of *tert*-butyl carbazate (1.0 mmol, 1.0 eq.) in DCM (10 mL) at 0 °C was added 3-(hexyloxy)benzoic acid (1.1 mmol, 1.1 eq.), DMAP (0.13 mmol, 0.13 eq.) and progressively DCC (1.2 mmol, 1.2 eq.). The mixture was then stirred at room temperature for 16 h, filtered on Celite^®^ and washed with DCM. The filtrate was then concentrated and the residue purified by chromatography (eluent: pentane/Et_2_O 2:1).

##### *Tert*-Butyl 2-(3-(hexyloxy)benzoyl)hydrazine-1-Carboxylate (11)

White powder (214 mg, 64% yield). ^1^H NMR (300 MHz, Acetone-*d*_6_) δ 9.41 (s, 1H), 7.95 (s, 1H), 7.51–7.44 (m, 2H), 7.38 (ddd, *J* = 8.1, 7.4, 0.7 Hz, 1H), 7.11 (ddd, *J* = 8.2, 2.5, 1.1 Hz, 1H), 4.05 (t, *J* = 6.5 Hz, 2H), 1.85–1.72 (m, 2H), 1.44 (s, 9H), 1.40–1.32 (m, 6H), 0.94–0.86 (m, 3H); ^13^C NMR (75 MHz, Acetone-*d*_6_) δ 167.17, 159.98, 156.69, 130.20, 120.17, 119.14, 113.57, 80.54, 68.57, 32.18, 29.79, 28.36, 26.30, 23.16, 14.27; MS (ESI) m/z = 337.3 [M+H]^+^, 359.3 [M+Na]^+^.

##### *Tert*-Butyl 2-(2,6-Difluoro-3-(Hexyloxy)benzoyl)hydrazine-1-Carboxylate (12)

White powder (136 mg, 52% yield). ^1^H NMR (300 MHz, Acetone-*d*_6_) δ 9.39 (s, 1H), 8.18 (s, 1H), 7.24 (td, *J* = 9.3, 5.2 Hz, 1H), 6.98 (td, *J* = 8.8, 2.1 Hz, 1H), 4.08 (t, *J* = 6.5 Hz, 2H), 1.84–1.72 (m, 2H), 1.46 (s, 9H), 1.40–1.27 (m, 6H), 0.96–0.86 (m, 3H); ^13^C NMR (75 MHz, Acetone-*d*_6_) δ 160.46, 153.72 (dd, *J* = 243.6, 6.0 Hz), 150.24 (dd, *J* = 251.5, 7.7 Hz), 144.75 (dd, *J* = 10.8, 3.4 Hz), 117.43 (dd, *J* = 9.3, 3.0 Hz), 115.09 (dd, *J* = 23.9, 19.5 Hz), 111.59 (dd, *J* = 22.8, 4.2 Hz), 80.65, 70.74, 32.21, 29.58, 28.38, 26.27, 23.23, 14.27; MS (ESI) m/z = 395.1 [M+Na]^+^.

#### 3.3.7. (viii) General Procedure for 3-(hexyloxy)benzohydrazide

To a solution of *tert*-butyl 2-(3-(hexyloxy)benzoyl)hydrazine-1-carboxylate (0.30 mmol, 1.0 eq.) in DCM (3 mL) was added TFA (12.0 mmol, 40 eq.). The mixture was stirred at room temperature for 24 h and then co-evaporated with toluene. The residue was retaken in sat. NaHCO_3_ solution, extracted with EtOAc, dried over Na_2_SO_4_, concentrated and purified by chromatography (eluent: EtOAc 100%).

##### 3-(Hexyloxy)benzohydrazide (13)

White powder (29 mg, 41% yield). ^1^H NMR (500 MHz, Methanol-*d*_4_) δ 7.37–7.29 (m, 3H), 7.08–7.02 (m, 1H), 4.00 (td, *J* = 6.5, 1.3 Hz, 2H), 1.83–1.68 (m, 2H), 1.54–1.41 (m, 2H), 1.40–1.33 (m, 4H), 0.96–0.88 (m, 3H); ^13^C NMR (126 MHz, Methanol-*d*_4_) δ 169.51, 160.72, 135.51, 130.67, 120.12, 119.10, 113.97, 69.18, 32.74, 30.29, 26.82, 23.67, 14.37; HRMS (ESI) m/z: calcd. for C_13_H_21_N_2_O_2_ [M+H]^+^ 237.1598, found 237.1600.

##### 2,6-Difluoro-3-(hexyloxy)benzohydrazide (14)

White powder (51 mg, 94% yield). ^1^H NMR (300 MHz, Methanol-*d*_4_) δ 7.17 (tdd, *J* = 9.2, 5.2, 2.0 Hz, 1H), 6.95 (dddd, *J* = 9.2, 8.7, 2.0, 0.9 Hz, 1H), 4.04 (td, *J* = 6.4, 2.4 Hz, 2H), 1.84–1.72 (m, 2H), 1.56–1.43 (m, 2H), 1.41–1.28 (m, 4H), 1.00–0.86 (m, 3H); ^13^C NMR (126 MHz, Methanol-*d*_4_) δ 162.26, 154.13 (dd, *J* = 243.2, 5.7 Hz), 150.66 (dd, *J* = 251.2, 7.5 Hz), 145.23 (dd, *J* = 10.8, 3.2 Hz), 117.81 (dd, *J* = 9.4, 3.1 Hz), 115.22 (dd, *J* = 23.7, 19.1 Hz), 111.80 (dd, *J* = 22.9, 4.2 Hz), 71.25, 32.67, 30.25, 26.68, 23.65, 14.35; HRMS (ESI) m/z: calcd. for C_13_H_19_F_2_N_2_O_2_ [M+H]^+^ 273.1409, found 273.1411.

### 3.4. Biological Evaluation

MICs were evaluated in Cation-adjusted Mueller-Hilton Broth (CaMHB) by the method of microdilution in liquid medium, which follows the CLSI (Performance Standards for Antimicrobial Susceptibility Testing. 31st ed. CLSI supplement M100., Wayne, PA: Clinical and Laboratory Standards Institute; 2021). The 0.5 MacFarland bacterial suspensions were made from colonies previously grown on blood agar plate (COS, Biomérieux^®^, Marcy-l’Étoile, France) in a saline solution (0.45% NaCl). Evaluated compounds were diluted in DMSO with a concentration of 5 mg/mL and then further diluted in CaMHB (1/100) before addition into 96-well microplates. MICs were carried out in triplicate and determined after 18 h of incubation at 37 °C. The median values were reported.

## 4. Conclusions

This study contributes to the knowledge on the important fluorinated benzamide pharmacophore for allosteric inhibition of *Sa*FtsZ. While the benzamide function was confirmed to be essential for reaching FtsZ inhibiting activity, the reason for increased activity due to fluorination has been scrutinized from the conformational viewpoint. The comparison of conformational analyses of 3-MBA and DFMBA unveiled the influence of the fluorine atoms on the conformation of the 2,6-difluorobenzamide by inducing a nonplanar conformation, rending easier for the ligand to adopt the known active conformation. Docking studies of the calculated preferential conformation shows results very close to the co-crystallized structure, indicating van der Walls interactions of the fluorine atoms with the residues Val203, Val297 and Asn263, while the benzamide functional group develops interactions with Val207, Leu209 and Asn263 via hydrogen bonds.

## Figures and Tables

**Table 1:** Minimum inhibitory concentrations of benzamides and analogues against *S. aureus* strains.

Compound	MIC (µg/mL)		
	ATCC29213 ^a^	SF8300 ^b^	ST20171643 ^c^
**9**, **10**, **13**, **14**	≥64		
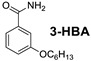	256	256	256
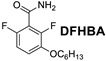	8	8	8

^a^: methicillin-sensitive *S. aureus*; ^b^: methicillin-resistant *S. aureus*; ^c^: daptomycin-resistant *S. aureus.*

## Data Availability

Data can be found in the manuscript and in the Supplementary Materials.

## Supplementary Materials

The following supporting information can be downloaded at: https://www.mdpi.com/article/10.3390/molecules28052055/s1. Figure S1: Conformational analysis of DFHBA and 3-HBA obtained as a result of variation of the torsion angle between the carboxamide and the phenyl group.
