# Supplementary material for: Importance of the 2,6-Difluorobenzamide Motif for FtsZ Allosteric Inhibition: Insights from Conformational Analysis, Molecular Docking and Structural Modifications
Source: Molecules. 2023 Feb 22;28(5):2055. doi: 10.3390/molecules28052055 (PMC10003973; doi:10.3390/molecules28052055)
Supplement: Supplementary file 1 [file molecules-28-02055-s001.zip › molecules-2220871-supplementary.pdf]

molecules

MDPI

Supplementary Materials

Importance of the 2,6-Difluorobenzamide Motif for FtsZ Allosteric Inhibition: Insights from Conformational Analysis, Molecular Docking and Structural Modifications

Thibaut Barbier [1], Oana Dumitrescu [2,3], Gérard Lina [2,3], Yves Queneau [1] and Laurent Soulère [1,*]

[1] Univ Lyon, INSA Lyon, Université Claude Bernard Lyon 1, CNRS, CPE-Lyon, ICBMS, UMR 5246, Institut de Chimie et de Biochimie Moléculaires et Supramoléculaires, Bâtiment Lederer, 1 Rue Victor Grignard, 69622 Villeurbanne, France

[2] Hospices Civils de Lyon, Hôpital de la Croix Rousse-Centre de Biologie Nord, Laboratoire de Bactériologie, Institut des Agents Infectieux, Grande Rue de la Croix Rousse, 69004 Lyon, France

[3] Team StaPath, CIRI, Centre International de Recherche en Infectiologie, Inserm, U1111, Université Claude Bernard Lyon 1, CNRS, UMR 5308, ENS de Lyon, 69007 Lyon, France

* Correspondence: laurent.soulere@insa-lyon.fr

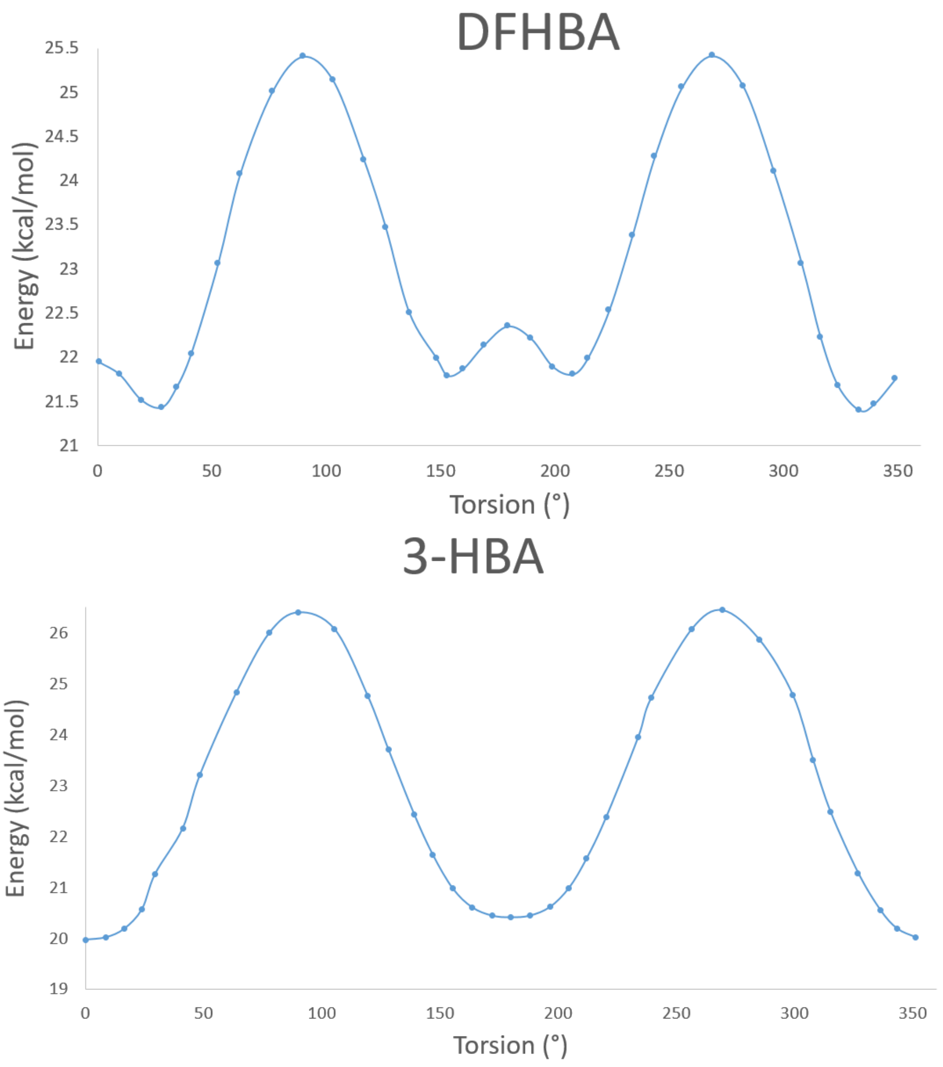

Figure S1. Conformational analysis of DFHBA and 3-HBA obtained as a result of variation of the torsion angle between the carboxamide and the phenyl group.
